# JOSD1 promotes proliferation and chemoresistance of head and neck squamous cell carcinoma under the epigenetic regulation of BRD4

**DOI:** 10.1186/s12935-021-02060-1

**Published:** 2021-07-14

**Authors:** Chao Jing, Dandan Liu, Qingchuan Lai, Linqi Li, Mengqian Zhou, Beibei Ye, Yue Wu, Hong Li, Kai Yue, Yansheng Wu, Yuansheng Duan, Xudong Wang

**Affiliations:** 1 Department of Maxillofacial and Otorhinolaryngological Oncology, Tianjin Medical University Cancer Institute and Hospital, National Clinical Research Center of Cancer, Tianjin, 300060 China; 2 Key Laboratory of Cancer Prevention and Therapy, Tianjin, 300060 China; 3 Tianjin’s Clinical Research Center for Cancer, Tianjin, 300060 China

**Keywords:** Head and neck squamous cell carcinoma, JOSD1, BRD4, Proliferation, Chemoresistance

## Abstract

**Background:**

Deubiquitinating enzymes (DUBs) play critical roles in various cancers by modulating functional proteins post-translationally. Previous studies have demonstrated that DUB Josephin Domain Containing 1 (JOSD1) is implicated in tumor progression, however, the role and mechanism of JOSD1 in head and neck squamous cell carcinoma (HNSCC) remain to be explored. In this study, we aimed to identify the clinical significance and function of JOSD1 in HNSCC.

**Methods:**

The Cancer Genome Atlas (TCGA) and Gene Expression Omnibus (GEO) databases were analyzed to find novel DUBs in HNSCC. Immunohistochemistry assay was performed to determine the expression of JOSD1 in our cohort of 42 patients suffered with HNSCC. Kaplan–Meier analysis was used to identify the correlation between JOSD1 and the prognosis of HNSCC patients. The regulation of BRD4 on JOSD1 was determined by using pharmacological inhibition and gene depletion. The in vitro and in vivo experiments were conducted to elucidate the role of JOSD1 in HNSCC.

**Results:**

The results of IHC showed that JOSD1 was aberrantly expressed in HNSCC specimens, especially in the chemoresistant ones. The overexpression of JOSD1 indicated poor clinical outcome of HNSCC patients. Moreover, JOSD1 depletion dramatically impaired cell proliferation and colony formation, and promoted cisplatin-induced apoptosis of HNSCC cells in vitro. Additionally, JOSD1 suppression inhibited the tumor growth and improved chemosensitivity in vivo. The epigenetic regulator BRD4 contributed to the upregulation of JOSD1 in HNSCC.

**Conclusions:**

These results demonstrate that JOSD1 functions as an oncogene in HNSCC progression, and provide a promising target for clinical diagnosis and therapy of HNSCC.

## Background

Head and neck cancer (HNC) ranks the sixth most common cancers worldwide, including cancers of oral cavity, pharynx, and larynx. Head and neck squamous cell carcinoma accounts (HNSCC) for about 90% of HNC [1–3], and most patients are diagnosed with advanced stages of HNSCC. Importantly, the 5-year overall survival rate of HNSCC patients remains less than 50% despite the improvement of therapeutic strategies [4]. Malignant proliferation and chemoresistance are major contributors to unfavorable prognosis in HNSCC [5]. Cisplatin-based regimen is the first-line treatment for patients with HNSCC [3, 6]. Therefore, to find novel and potential targets has been an imperative need to improve survival and life quality of HNSCC patients.

Ubiquitination is one of the most important posttranslational modifications, and mainly mediates degradation of protein [7]. Deubiquitinating enzymes (DUBs) could reverse ubiquitination process by removing ubiquitin from the substrates, which improves the stability of substrates and prevents protein degradation mediated by proteasome [8]. DUBs families are classified into two types, cysteine proteases (including USPs, UCHs, OTUs, MJDs, MINDYs, and ZUP1 family) and zinc-dependent metalloproteinases (JAMM family) [9, 10]. Of note, MJDs is the smallest DUBs family, only composed of Ataxin-3, Ataxin-3L, JOSD1, JOSD2. Among them, Ataxin-3 has been well studied and could play crucial roles in autophagy and tumor progression [11, 12]. JOSD1, which only contains a highly conserved Josephin domain, is located in chromosome 22 q13.1 [13]. Previous studies have indicated that JOSD1 stabilizes the target proteins by cleaving the K48 ubiquitin chains [14, 15]. In gynaecological cancer, JOSD1 promotes acquired chemoresistance by stabilizing MCL1 [14]. Besides, as a membrane-associated DUB, JOSD1 could regulate membrane dynamics, cell motility, and endocytosis [16]. However, the role of JOSD1 in HNSCC remains to be explored.

An increasing amount of evidence have showed that epigenetic dysregulation, such as histone modifications, contributes to tumorigenesis and progression [17]. The bromodomain and extra-terminal domain (BET) proteins are critical epigenetic readers and transcriptional coactivators, selectively bind to acetylated lysine residues of histone H3 and H4 on chromatin to regulate gene expression [18]. As a well-studied member of BET family, bromodomain-containing protein 4 (BRD4) could recruit mediator, transcriptional factors, the transcription elongation factor P-TEFb or other histone modifiers to facilitate transcriptional activation of target genes [19, 20]. Recent studies showed that BRD4 is implicated in tumor malignancies [19–21], therefore, serves as a promising therapeutic target. The small molecule inhibitor JQ1 could suppress tumorigenesis and malignant progression by targeting BRD4 [21–23].

In this study, we demonstrate that JOSD1 was overexpressed in HNSCC under the epigenetic regulation of BRD4. The elevated expression of JOSD1 correlated positively with chemoresistance and predicted poor prognosis of patients with HNSCC. Furthermore, JOSD1 silencing could inhibit proliferation and enhance chemosensitivity of HNSCC cells in vitro and in vivo. Together, these results shed light on the critical role of JOSD1 in HNSCC progression and provide a promising target to overcome resistance to chemotherapy in HNSCC.

## Materials and methods

### Bioinformatics analysis

The online software Gene Expression Profiling Interactive Analysis (GEPIA, gepia.cancer-pku.cn) and GEO2R were used to analyze the levels of DUBs in HNSCC based on The Cancer Genome Atlas (TCGA) and Gene Expression Omnibus (GEO) databases, respectively.

### Cell culture

Human HNSCC cell lines SCC25 and TSCCA were maintained in DMEM/F-12 or RPMI1640 (Invitrogen, Camarillo, CA, USA) supplemented with 10% FBS (Gibco) and 1% penicillin/streptomycin under a humidified atmosphere (37 °C, 5% CO_2_). All HNSCC cells were authorized by STR analysis. HNSCC CDDP-resistant cell lines (CDDP-R TSCCA and CDDP-R SCC25) were established by continuous exposure to CDDP at increasing concentrations over 6 months.

### Antibodies and reagents

Antibodies used for immunoblotting (IB) and immunohistochemistry (IHC) were as follows: JOSD1, ORIGENE (Rockville, MD, USA), TA502210 (IB, 1:1000; IHC, 1:100); BRD4, Active Motif (Carlsbad, CA, USA), #39909 (IB, 1:1000; IHC, 1:500); cleaved PARP, Cell Signaling Technology (Danvers, MA, USA), #9542 (IB, 1:1000); cleaved Caspase-3, Cell Signaling Technology, #9661(IB, 1:1000; IHC, 1:400); Ki-67, Cell Signaling Technology, #9449 (IHC, 1:500); GAPDH, Santa Cruz (Dallas, Texas, USA), sc-365062 (IB, 1:5000). BRD4 inhibitor JQ1 was purchased from Selleck (Shanghai, China).

### Plasmids and transduction

The BRD4-overexpressing plasmid was purchased from Vigenebio (Shandong, China). The shRNAs sequences targeting shJOSD1#1, shJOSD1#2 and shBRD4 were 5′-GAGCGAGCTCAGGAAGTTTCT-3′, 5′-GGTGGTACCAGAAGAGGTAGA-3′ and 5′-CAGTGACAGTTCGACTGATGA-3′ respectively, which were cloned into the pSIH-H1-puro lentiviral vector. The stable clones were exposed to puromycin (1–2 μg/ml) for at least one week after lentivirus infection. The knockdown effects of specific shRNAs on JOSD1 and BRD4 were confirmed by using western blot assay.

### MTT assay

MTT assay was used to examine cell proliferation and viability according to the protocol of manufacturer. Briefly, cells were seeded into 96-well plates and incubated at 37 °C overnight for adherence. The MTT crystals were dissolved in DMSO, and then the absorbance at 490 nm was measured by using a microplate reader (Model 680, Bio-Rad Laboratories Ltd., Hercules, California, USA).

### Clonogenicity assay

HNSCC cells were seeded into 6-well plates (1000 cells/well) and incubated at 37 °C for 14 days. Then, the cells were washed twice with PBS, fixed and stained with 0.1% crystal violet. Colonies with > 50 cells were counted under an inverted microscope (DMI6000B, Leica).

### Quantitative real-time PCR (qRT-PCR)

Total RNAs were extracted using TRIzol (Invitrogen, Carlsbad, California, USA) according to the manufacturer’s instructions. Reverse transcription and qRT-PCR were performed using the PrimeScript™ RT Master Mix and SYBR Premix Ex Taq™ II (Takara, Shiga, Japan) according to the manufacturer’s instructions, respectively. The expressions of target genes were normalized to the expression of glyceraldehyde-3-phosphate dehydrogenase (GAPDH) and evaluated by using the 2^−ΔΔCt^ method. The sequences of primers used were as follows:

JOSD1, forward: 5′-GGGATACGCTGCAAGAGATTT-3′;

JOSD1, reverse: 5′-CCATGACGTTAGTGAGGGCA-3′;

BRD4, forward: 5′-AGCAGCAACAGCAATGTGAG-3′;

BRD4, reverse: 5′-GCTTGCACTTGTCCTCTTCC-3′;

GADPH, forward: 5′-TGCACCACCAACTGCTTAGC-3′;

GAPDH, reverse: 5′-GGCATGGACTGTGGTCATGAG-3′.

### Western blot

Western blot analysis was employed to assess protein expression. Cells were harvested and lysed in cell lysates buffer supplemented with protease and phosphatase inhibitors (Solarbio Science & Technology Co., Ltd., Beijing, China). Protein concentrations were determined using Micro BCA Protein Assay kit (Thermo Fisher Scientific, Inc.). The same amounts of protein samples (20–30 μg) were separated by 10% SDS-PAGE and were transferred onto polyvinylidene difluoride (PVDF) membranes (Merck Millipore). After blocking with 5% non-fat milk, the membranes were incubated with primary antibodies overnight at 4 °C, and then incubated with secondary antibodies (1:5000) at room temperature for another 1 h. The abundances of target proteins were detected by using ECL chemiluminescence kit (Cell Signaling Technology).

### Flow cytometry

Annexin V-FITC/PI Apoptosis Assay Kit (BD, Franklin Lakes, NJ, USA) was used to evaluate cell apoptotic rate according to the manufacturer’s instruction. Briefly, HNSCC cells treated with CDDP were harvested and washed three times with PBS. Then, the cells were stained with Annexin V-FITC and PI at room temperature in the dark for 15 min. The apoptotic rate of treated cells was measured on the FACS Canto II (BD).

### Immunohistochemistry (IHC)

The paraffin-embedded tumor tissue sections were deparaffinized, rehydrated and incubated with primary antibodies against JOSD1, BRD4, Ki67, Cleaved-Caspase3 overnight at 4 °C. After incubation with secondary antibodies for 30 min at room temperature, sections were visualized by using diaminobenzidine (DAB) staining.

### Animal experiment

Five-week-old male BALB/C nude mice were maintained in a specific pathologic free (SPF) environment. 3 × 10^6^ TSCCA cells expressing shJOSD1 or shNC were inoculated subcutaneously into the flanks of mice. Then, the mice bearing xenografts were administered with CDDP (2.5 mg/kg) or saline three times a week (5 mice per group). Finally, mice were sacrificed (euthanized with a 30% CO_2_ flow rate in a darkened chamber for 2 min) and the tumors were harvested for IHC detection.

### Statistical analysis

All data from at least three independent experiments were presented as mean ± SD and analyzed by using Student's t-test. The differences with P < 0.05 (two-sided) were considered statistically significant. All statistical analyses were performed using GraphPad Prism 6 or SPSS 21.0 software.

## Results

### Aberrant expression of JOSD1 indicates poor prognosis of HNSCC patients

**Fig. 1:**
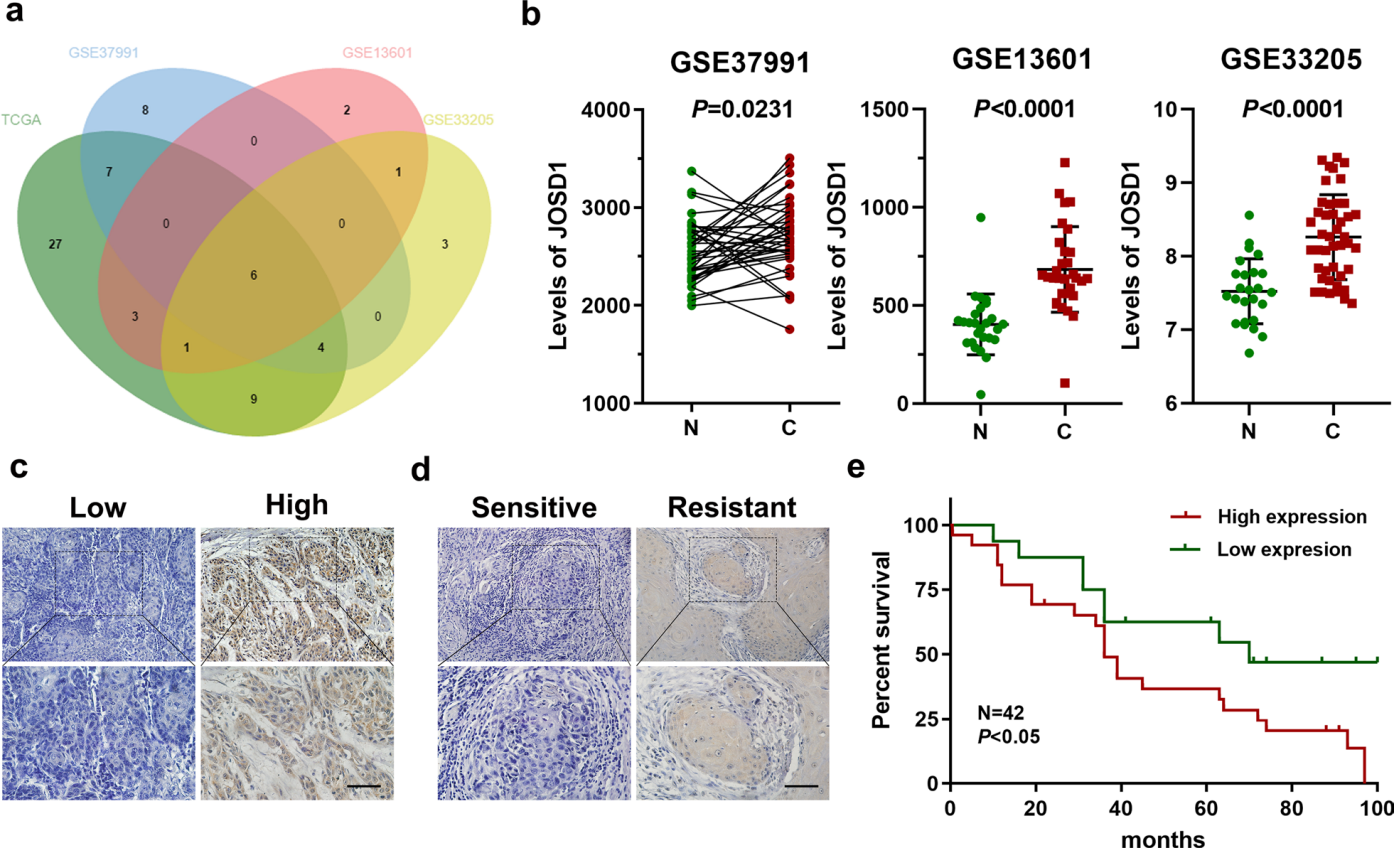
Elevated JOSD1 indicates unfavorable prognosis in HNSCC. **a** The Venn diagrams based on TCGA and GEO databases identified significantly upregulated DUBs in HNSCC, including JOSD1, PSMD14, SENP5, TNFAIP3, UCK2, UFD1L. **b** The expression of JOSD1 was increased in HNSCC compared to normal tissues in three independent GEO datasets (GSE13601, GSE33205, GSE37991). N, normal tissue. C, cancerous specimen. Data, mean ± SD. **c** The representative images of IHC staining to assess the expression of JOSD1 in HNSCC tissues. Scale bar, 50 μm. **d** The IHC results showed that JOSD1 was overexpressed in chemoresistant HNSCC samples relative to the sensitive ones. Scale bar, 50 μm. **e** Kaplan–Meier survival curve revealed that the high expression of JOSD1 predicted poor prognosis of patients with HNSCC

To identify differentially expressed DUBs in HNSCC, the levels of 115 DUBs were analyzed based on TCGA database and three GEO datasets (GSE37991, GSE13601, GSE33205). Then, we found that six DUBs including JOSD1 were significantly elevated in HNSCC samples in each cohort (Fig. 1a, b). Subsequently, IHC staining was performed to detect the expression of JOSD1 in 42 HNSCC tissues from our cohort, and the results showed that JOSD1 was located in the cytoplasm and cytomembrane (Fig. 1c). Further analysis indicated that JOSD1 significantly correlated with T stage, clinical stage, and chemoresistance in HNSCC (Table 1). Compared with sensitive specimens, JOSD1 expression was dramatically increased in chemoresistant tissues (Fig. 1d), indicating a potential role of JOSD1 in HNSCC chemoresistance. Moreover, Kaplan–Meier survival curve revealed that HNSCC patients with higher JOSD1 level exhibited worse prognosis (Fig. 1e). Collectively, these data suggest that JOSD1 is aberrantly expressed in HNSCC and predicts poor prognosis.

**Table 1:** JOSD1 Expression and Clinicopathological Features of HNSCC

Clinicopathological Features	JOSD1 expression		Total	*P* value
	Low	High		
Gender				
Male	13 (38.2%)	21 (61.8%)	34	0.256
Female	5 (62.5%)	3 (37.5%)	8	
Age				
≥ 60	9 (39.1%)	14 (60.9%)	23	0.591
< 60	9 (47.4%)	10 (52.6%)	19	
Smoke				
Yes	10 (40.0%)	15 (60.0%)	25	0.650
No	8 (47.1%)	9 (52.9%)	17	
Drink				
Yes	6 (30.0%)	14 (70.0%)	20	0.108
No	12 (54.5%)	10 (45.5%)	22	
T stage				
T1–T2	14 (58.3%)	10 (41.7%)	24	0.019*
T3–T4	4 (22.2%)	14 (77.8%)	18	
N stage				
N0	12 (52.2%)	11 (47.8%)	23	0.179
N1–3	6 (31.6%)	13 (68.4%)	19	
Clinical stage				
I–II	11 (57.9%)	7 (42.1%)	18	0.038*
III–IV	7 (21.7%)	17 (78.3%)	24	
Histological grade				
G1	5 (35.7%)	9 (64.3%)	14	0.532
G2	11 (52.4%)	10 (47.6%)	21	
G3	2 (28.6%)	5 (71.4%)	7	
Resistance				
PR	8 (72.7%)	3 (27.3%)	11	0.048*
PD/SD	10 (32.3%)	21 (67.7%)	31	

The result was analyzed by the Pearson χ2 test. *P* values with signifirance were shown as asterisk

^*^ *P* < 0.05

### BRD4 regulates JOSD1 in HNSCC

**Fig. 2:**
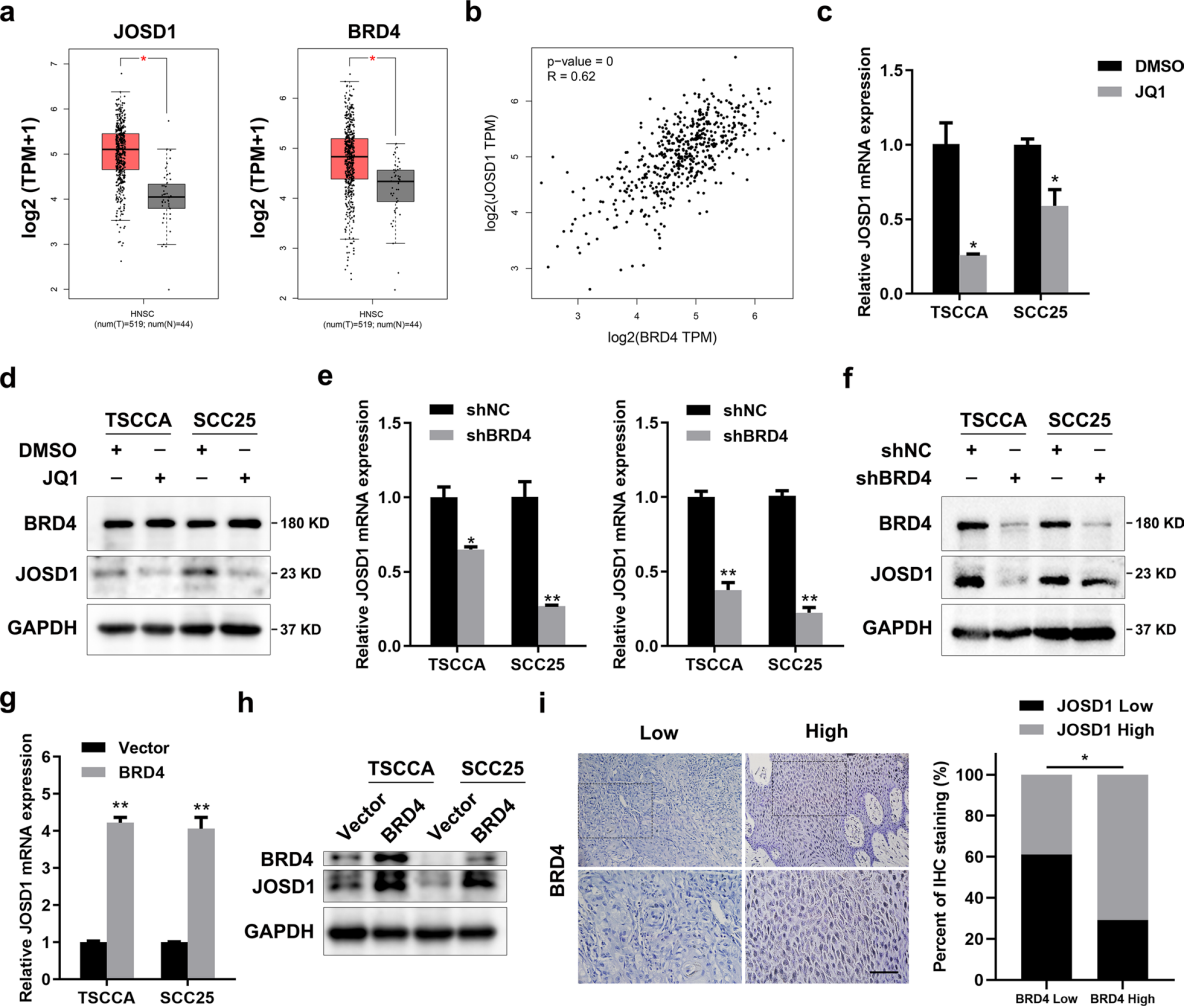
BRD4 inhibition downregulates JOSD1 in HNSCC. **a** TCGA-HNSCC database analysis showed that both JOSD1 and BRD4 were overexpressed in HNSCC tissues. **b** BRD4 correlated positively with JOSD1, which was analyzed by GEPIA. **c, d** The effect of JQ1 on JOSD1 was detected by using qRT-PCR and western blot assay. The dosage of JQ1, 250 nM. **e, f** The mRNA and protein expression of BRD4 and JOSD1 were measured in the HNSCC cells expressing shBRD4. **g, h** The effect of BRD4 overexpression of JOSD1 was measured by using qPCR and western blot assays. **i** The representative images of IHC staining of BRD4 and the analysis of the correlation between BRD4 and JOSD1 in HNSCC tissues. Scale bar, 50 μm. Data in this figure, mean ± SD, *P < 0.05, **P < 0.01. TPM, transcripts per million

Next, the cause of increased JOSD1 was explored. Through the analysis of TCGA-HNSCC database, we validated that JOSD1 and BRD4 were both overexpressed in HNSCC tissues compared with normal tissues (Fig. 2a). Then, we further observed a markedly positive correlation between JOSD1 and BRD4 (Fig. 2b). To assess the effect of BRD4 on JOSD1, a small molecule inhibitor JQ1 was used by inhibiting the activity of BRD4. The results showed that the administration of JQ1 to HNSCC cells strikingly downregulated JOSD1 in mRNA and protein level (Fig. 2c, d). Besides, we found that knockdown of BRD4 by shRNA, resembling the effect of JQ1, inhibited the expression of JOSD1 (Fig. 2e, f), while BRD4 overexpression significantly upregulated JOSD1 in HNSCC cells (Fig. 2g, h), suggesting that BRD4 affected JOSD1 expression at the transcriptional level. Moreover, the analysis of IHC staining of BRD4 and JOSD1 in HNSCC tissues further indicated a positive correlation between them (Fig. 2i). Taken together, these evidences illustrate that BRD4, as an epigenetic modulator, enhances the expression of JOSD1 by facilitating its transcription activation.

### JOSD1 depletion inhibits resistance to cisplatin and cell growth in HNSCC in vitro

**Fig. 3:**
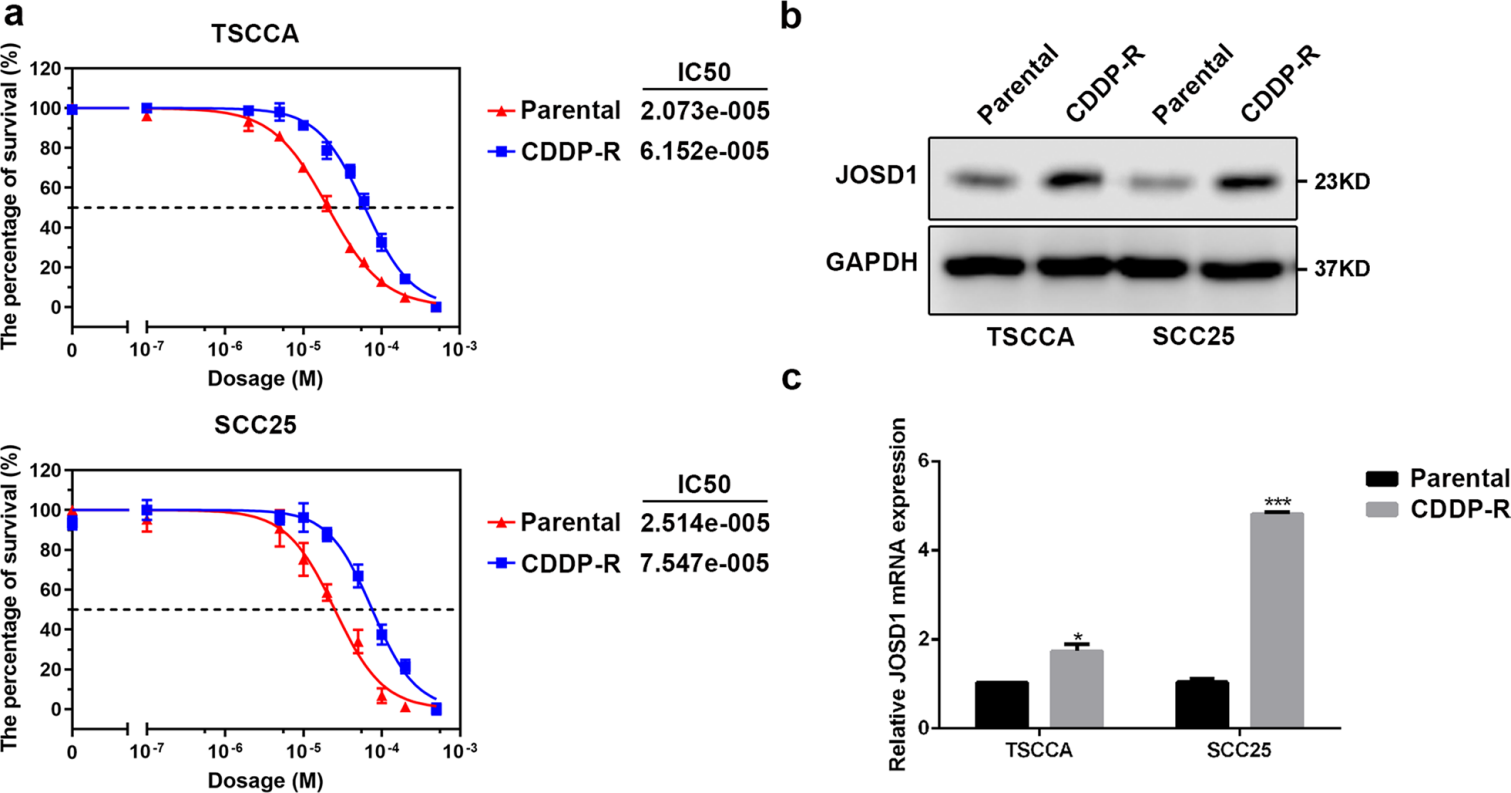
JOSD1 level is increased in acquired chemoresistant HNSCC cells. **a** The IC50 values of cisplatin in parental and cisplatin-resistant HNSCC cells were determined by using MTT assay. **b, c** The protein and mRNA levels of JOSD1 were enhanced in resistance HNSCC cells compared with the parental cells. GADPH was used as a loading control. Data in this figure, mean ± SD, *P < 0.05, ***P < 0.001. CDDP-R, cisplatin-resistant

**Fig. 4:**
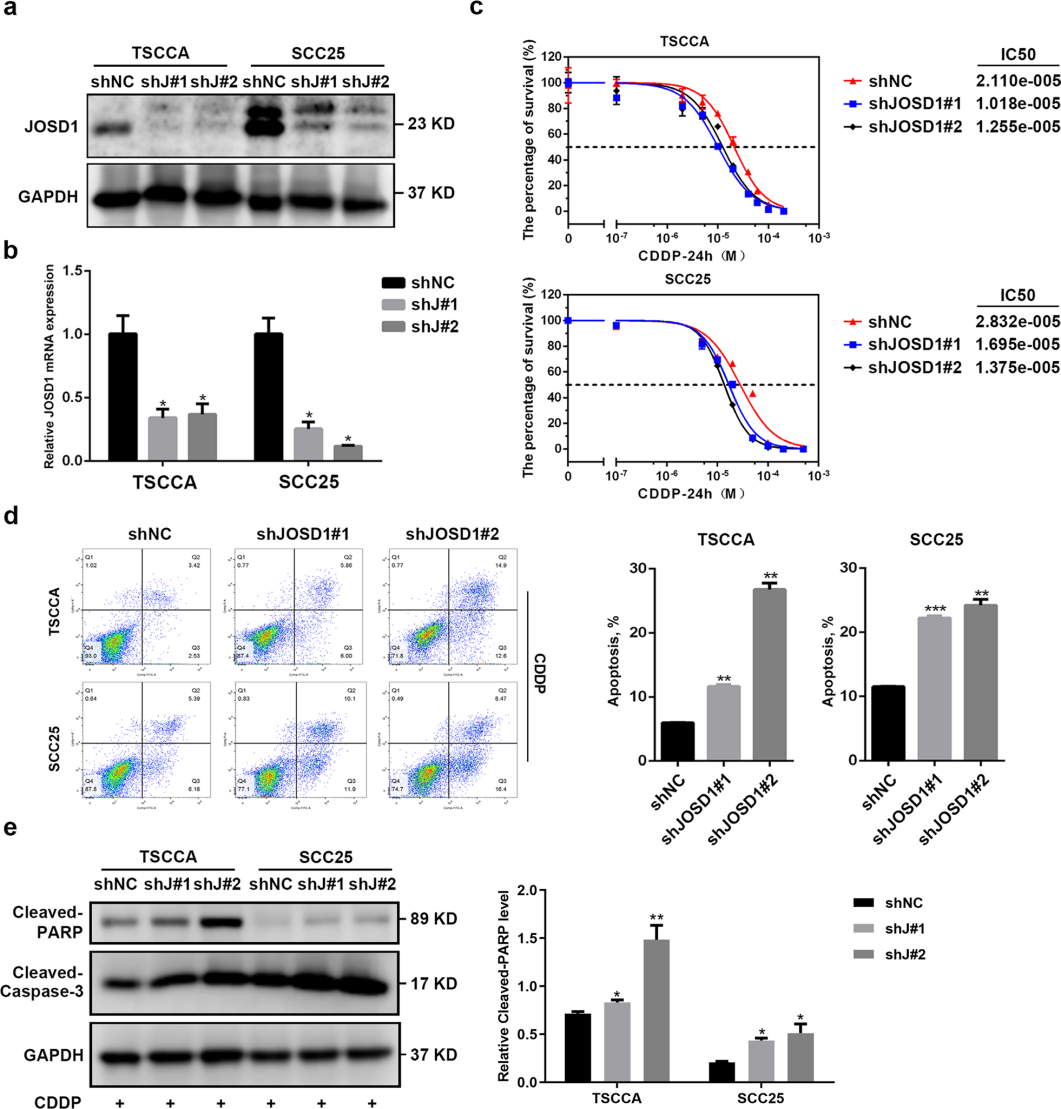
HNSCC cells with reduced JOSD1 level become sensitive to cisplatin treatment. **a**, **b** The protein and mRNA expression of JOSD1 were measured in the HNSCC cells expressing control (shNC) and JOSD1 shRNAs (shJ#1 and shJ#2). **c** The HNSCC cells expressing shNC or shJOSD1 were treated with CDDP at various concentrations for 24 h. Then, the IC50 values of CDDP were detected by using MTT assay. **d** Knockdown of JOSD1 significantly promoted cisplatin-induced apoptosis, which was confirmed by flow cytometry. **e** The abundance of cleaved PARP and cleaved Caspase-3 was measured by immunoblotting in shJOSD1-expressing HNSCC cells exposed to cisplatin. Data in this figure, mean ± SD, *P < 0.05, **P < 0.01, ***P < 0.001. CDDP, cisplatin

**Fig. 5:**
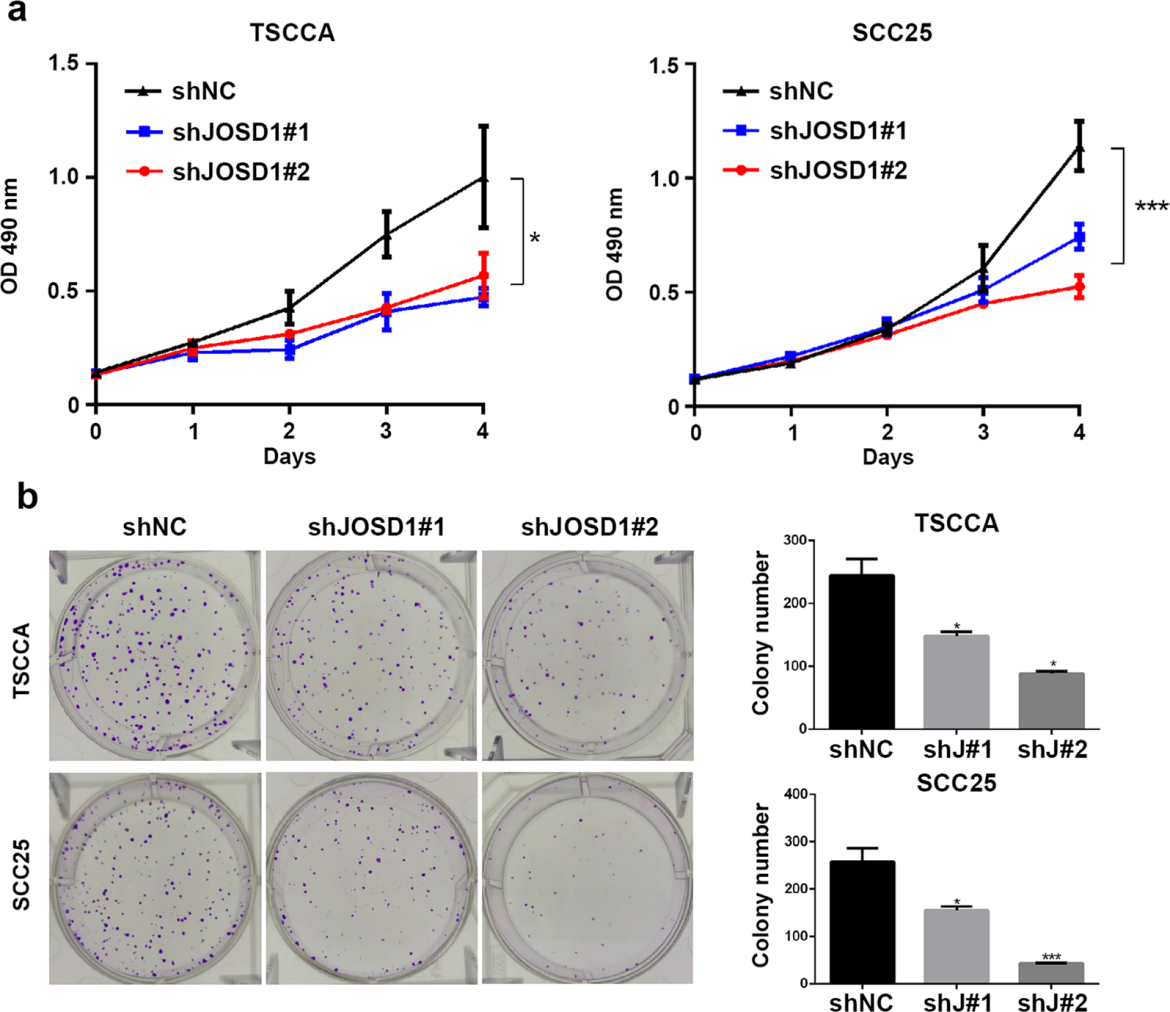
JOSD1 depletion inhibits HNSCC cell proliferation in vitro. **a** MTT assay was conducted to determine growth curve of HNSCC cells expressing JOSD1 shRNAs. **b** The results of clonogenicity assay indicated that JOSD1 silencing impaired the capacity of colony formation of HNSCC cells in vitro. Data in this figure, mean ± SD, *P < 0.05, ***P < 0.001

Then, the effect of JOSD1 in HNSCC was assessed through a series of functional experiments. We firstly established two cisplatin-resistant (CDDP-R) subclones in HNSCC TSCCA and SCC25 cell lines respectively, and observed that the IC50 values of CDDP in CDDP-R cells were obviously increased in relative to the parental cells (Fig. 3a). As shown in Fig. 3b, c, both protein and mRNA levels of JOSD1 were significantly elevated in CDDP-R HNSCC cells, suggesting that JOSD1 may be involved in chemoresistance of HNSCC. Then, we used two distinct shRNAs to knockdown the expression of JOSD1 (Fig. 4a, b). The results of MTT assay showed that the IC50 value of CDDP was dramatically reduced in the HNSCC cells expressing JOSD1 shRNAs, indicating that JOSD1 knockdown could sensitize HNSCC cells to CDDP (Fig. 4c). Meanwhile, we found that depletion of JOSD1 led to a significant increase of apoptosis rate of HNSCC cells treated with CDDP by using flow cytometry (Fig. 4d). Additionally, we also evaluated the level of apoptosis-related proteins in JOSD1-depleted HNSCC cells after CDDP treatment. As shown in Fig. 4e, the expressions of cleaved-PARP and cleaved-Caspase-3 both elevated in TSCCA and SCC25 cells expressing shJOSD1 compared with the negative control, suggesting that JOSD1 silencing promoted CDDP-induced apoptosis in HNSCC cells. In addition to chemoresistance, malignant growth is another main cause of unfavorable prognosis of HNSCC patients. We found that the growth was impeded in JOSD1-deficient TSCCA cells compared with the control cells (Fig. 5a). Similar inhibitory effect was also observed in SCC25 cells with deficient JOSD1 (Fig. 5a). Moreover, reduced level of JOSD1 impaired the capacity of colony formation in both TSCCA and SCC25 cells (Fig. 5b). Together, these results above corroborate that JOSD1 strengthens proliferation and chemoresistance in HNSCC cells.

### Knockdown of JOSD1 attenuates tumor growth and improves chemosensitivity of HNSCC cells in vivo

**Fig. 6:**
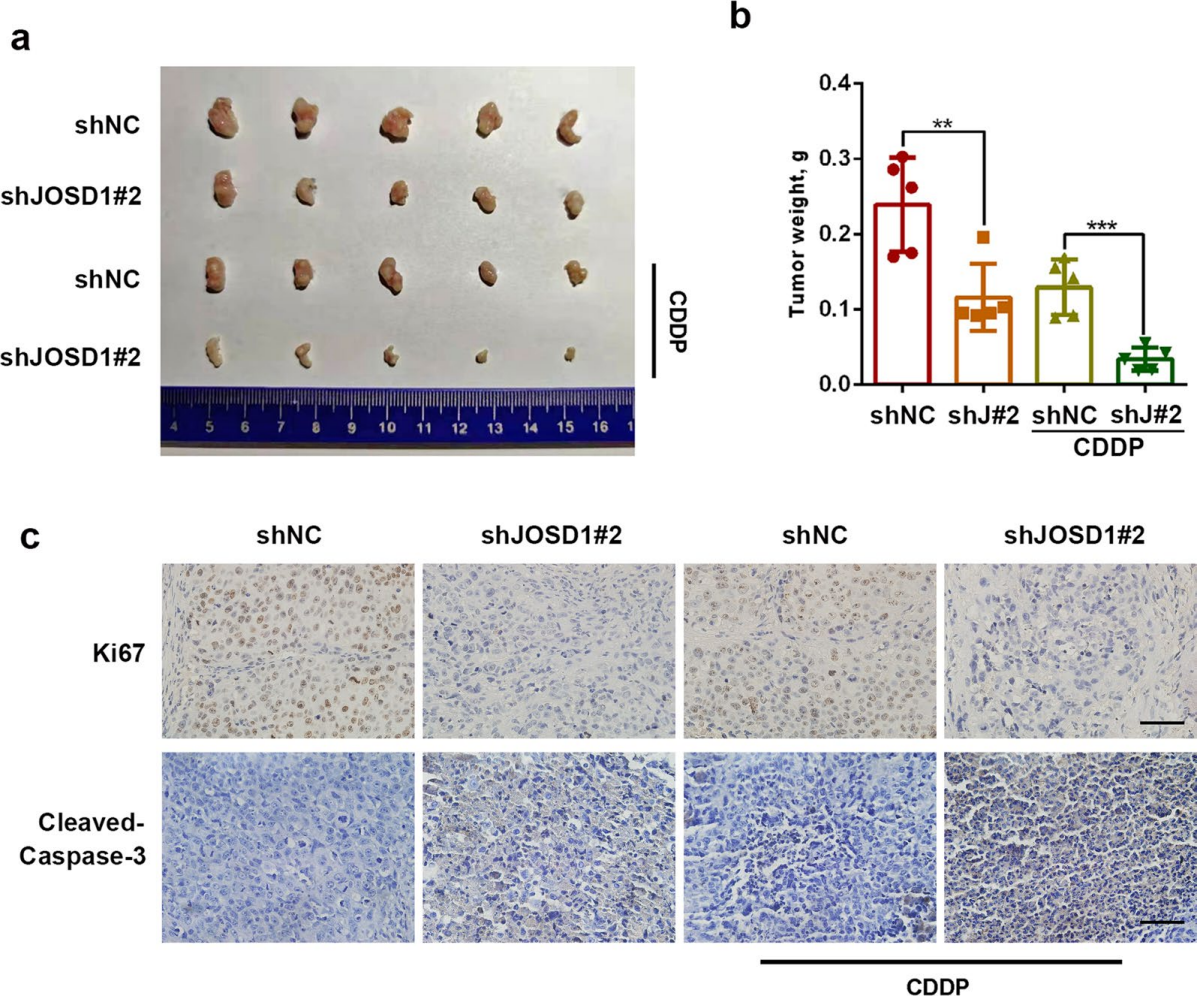
JOSD1 knockdown impedes tumor growth and increases chemosensitivity of HNSCC cells in vivo. **a**, **b** The representative photos and weight of xenografts generated from JOSD1-depleted TSCCA cells and the negative control (n = 5) under indicated treatments. Data, mean ± SD, **P < 0.01, ***P < 0.001. **c** The levels of Ki67 and Cleaved Caspase-3 in indicated groups were measured by using IHC staining. Scale bar, 50 μm

Finally, we further detected the in vivo role of JOSD1 in HNSCC by using xenograft model. The results showed that the xenografts generated from JOSD1-silenced TSCCA cells weighed significantly less than that generated from negative control cells (Fig. 6a, b), revealing an oncogenic role of JOSD1 in tumor growth of HNSCC. Additionally, compared with the control group, the tumor weight of shJOSD1 group showed a more decrease under the same dose of CDDP (Fig. 6a, b). As shown in Fig. 6c, the positive rate of Ki67 in JOSD1-depleted group was obviously reduced relative to the negative control group. Meanwhile, JOSD1 silencing combined with CDDP treatment extremely enhanced the positive staining of cleaved Caspase-3 compared with the other three groups (Fig. 6c), which indicated that JOSD1 knockdown could improve the effect of CDDP for treating HNSCC in vivo*.* Taken together, these results demonstrate that JOSD1 could serve as a critical therapeutic target in HNSCC.

## Discussion

Ubiquitin proteasome system (UPS) plays an important role in maintaining the stability and function of proteins and thus regulates a variety of biological activities. Previous studies have demonstrated that protein degradation is mainly mediated by UPS [24]. As a key component of UPS, DUBs participate in the regulation of cell cycle, apoptosis, proliferation and other behaviors [25]. Importantly, the dysregulation of DUBs leads to the progression of numerous human malignancies, including digestive cancers [26], breast cancer [27–29], OSCC [30] and HNSCC [31]. In this study, we analyzed public databases online and identified several significantly upregulated DUBs including JOSD1. JOSD1 is a member of the smallest DUBs family-MJDs family, containing a highly conserved Josephin domain [14, 15, 32]. The genetic aberrations of JOSD1 have presented in multiple cancers, particularly in melanoma, uterine, bladder, and ovarian cancer [13]. In the gynaecological cancer, JOSD1 is the most upregulated DUB in the development of chemoresistance and is considered as an ideal therapeutic target [14]. Through bioinformatic analysis and IHC detection, we found that JOSD1 was overexpressed in HNSCC and correlated with T stage, clinical stage and chemoresistance. Moreover, the HNSCC patients with JOSD1 overexpression had a shorter overall survival. These results suggest that JOSD1 could be a potential risk factor to guide treatment and predict prognosis in HNSCC.

So far, cisplatin-based treatment is still the standard regimen for HNSCC patients. However, intrinsic and acquired resistance to cisplatin is a major challenge to achieve a long-term curative effect and results in early recurrence and metastasis [33–35]. Therefore, there is an urgent need to find efficient means to improve cisplatin sensitivity. In this study, we detected the expression of JOSD1 in cisplatin-resistant cells lines and identified a high level of JOSD1 in CDDP-R TSCCA and CDDP-R SCC15 cells. Then, we observed that the IC50 of CDDP in JOSD1-silenced HNSCC cells was much lower than that in the control group. Moreover, with the treatment of CDDP, JOSD1 knockdown could increase expression of apoptosis-related proteins and promote cell apoptosis rate. The in vivo experiment also confirmed the promoting effect of JOSD1 on chemoresistance in HNSCC. In addition to chemoresistance, DUBs also participate in other malignant activities such as proliferation. For instance, USP10 and USP21 could promote hepatocellular carcinoma and non-small-cell lung cancer proliferation, respectively [36, 37]. In our study, JOSD1 knockdown could inhibit the growth and colony formation of TSCCA and SCC25 cells, and suppress the tumor growth in vivo. Furthermore, we found the high expression of Ki67 in JOSD1-depleted xenografts, indicating that JOSD1 could accelerate tumor proliferation in HNSCC. With those, we believe that JOSD1 could serve as a promising target to improve the clinical treatment of HNSCC.

Pioneering studies have revealed that BRD4 is frequently overexpressed in multiple cancers including HNSCC [19, 38–40], and promotes tumorigenesis, cancer cell proliferation, metastasis and drug resistance by elevating the expression of cancer drivers [41–44]. In this study, pairwise correlation analysis showed a remarkably positive correlation between BRD4 and JOSD1, indicating BRD4 may regulate JOSD1 expression. With the inhibition of BRD4 by using JQ1 and shRNA, the protein and mRNA expression of JOSD1 were both reduced. Consequently, the increase of JOSD1 in HNSCC tissues and resistant HNSCC cells may be partially dependent on the epigenetic regulation of BRD4, which also further enriches BRD4-mediated mechanisms of proliferation and chemoresistance. However, the specific mechanism of BRD4 regulating JOSD1 remains to be further explored in the further.

## Conclusion

In summary, these data demonstrate that JOSD1 upregulation in HNSCC positively correlated with poor prognosis of patients, uncovering the clinical prognostic significance of JOSD1. Under the regulation of BRD4, JOSD1 is a key driver of cell growth and chemoresistance in vitro and in vivo. Although more details of JOSD1 in HNSCC progression remain elusive, we based on these results believe that this DUB could be a promising candidate in future clinical practice of HNSCC.

## Data Availability

The datasets used and/or analyzed during the current study are available from the corresponding author on reasonable request.

## Abbreviations

HNSCC: Head and neck squamous cell carcinoma
HNC: Head and neck cancer
DUB: Deubiquitinating enzyme
TCGA: The Cancer Genome Atlas
CDDP: Cisplatin
STR: Short tandem repeat
FBS: Fetal bovine serum
IC50: Half maximal inhibitory concentration
UPS: Ubiquitin proteasome system

## References

[1] Bray F, Ferlay J, Soerjomataram I, Siegel RL, Torre LA, Jemal A (2018). Global cancer statistics 2018: GLOBOCAN estimates of incidence and mortality worldwide for 36 cancers in 185 countries. CA Cancer J Clin.

[2] Siegel RL, Miller KD, Jemal A (2020). Cancer statistics, 2020. CA Cancer J Clin.

[3] Johnson DE, Burtness B, Leemans CR, Lui VWY, Bauman JE, Grandis JR (2020). Head and neck squamous cell carcinoma. Nat Rev Dis Primers.

[4] Cohen EEW, Bell RB, Bifulco CB, Burtness B, Gillison ML, Harrington KJ, Le Q-T, Lee NY, Leidner R, Lewis RL (2019). The society for immunotherapy of cancer consensus statement on immunotherapy for the treatment of squamous cell carcinoma of the head and neck (HNSCC). J Immunother Cancer.

[5] Méry B, Rancoule C, Guy J-B, Espenel S, Wozny A-S, Battiston-Montagne P, Ardail D, Beuve M, Alphonse G, Rodriguez-Lafrasse C (2017). Preclinical models in HNSCC: a comprehensive review. Oral Oncol.

[6] Pendleton KP, Grandis JR (2013). Cisplatin-based chemotherapy options for recurrent and/or metastatic squamous cell cancer of the head and neck. Clin Med Insights Ther.

[7] Mevissen TET, Komander D (2017). Mechanisms of deubiquitinase specificity and regulation. Annu Rev Biochem.

[8] Mennerich D, Kubaichuk K, Kietzmann T (2019). DUBs, hypoxia, and cancer. Trends Cancer.

[9] Clague MJ, Urbé S, Komander D (2019). Breaking the chains: deubiquitylating enzyme specificity begets function. Nat Rev Mol Cell Biol.

[10] Li Y, Shi F, Hu J, Xie L, Bode AM, Cao Y (2019). The role of deubiquitinases in oncovirus and host interactions. J Oncol.

[11] Herzog LK, Kevei É, Marchante R, Böttcher C, Bindesbøll C, Lystad AH, Pfeiffer A, Gierisch ME, Salomons FA, Simonsen A (2020). The Machado-Joseph disease deubiquitylase ataxin-3 interacts with LC3C/GABARAP and promotes autophagy. Aging cell.

[12] Sacco JJ, Yau TY, Darling S, Patel V, Liu H, Urbé S, Clague MJ, Coulson JM (2014). The deubiquitylase Ataxin-3 restricts PTEN transcription in lung cancer cells. Oncogene.

[13] Zeng C, Zhao C, Ge F, Li Y, Cao J, Ying M, Lu J, He Q, Yang B, Dai X (2020). Machado-Joseph deubiquitinases: from cellular functions to potential therapy targets. Front Pharmacol.

[14] Wu X, Luo Q, Zhao P, Chang W, Wang Y, Shu T, Ding F, Li B, Liu Z (2020). JOSD1 inhibits mitochondrial apoptotic signalling to drive acquired chemoresistance in gynaecological cancer by stabilizing MCL1. Cell Death Differ.

[15] Wang X, Zhang L, Zhang Y, Zhao P, Qian L, Yuan Y, Liu J, Cheng Q, Xu W, Zuo Y (2017). JOSD1 negatively regulates type-i interferon antiviral activity by deubiquitinating and stabilizing SOCS1. Viral Immunol.

[16] Seki T, Gong L, Williams AJ, Sakai N, Todi SV, Paulson HL (2013). JosD1, a membrane-targeted deubiquitinating enzyme, is activated by ubiquitination and regulates membrane dynamics, cell motility, and endocytosis. J Biol Chem.

[17] Jones PA, Issa J, Baylin S (2016). Targeting the cancer epigenome for therapy. Nat Rev Genet.

[18] Zhu Z, Song J, Guo Y, Huang Z, Chen X, Dang X, Huang Y, Wang Y, Ou W, Yang Y (2020). LAMB3 promotes tumour progression through the AKT-FOXO3/4 axis and is transcriptionally regulated by the BRD2/acetylated ELK4 complex in colorectal cancer. Oncogene.

[19] Wu Y, Wang Y, Diao P, Zhang W, Li J, Ge H, Song Y, Li Z, Wang D, Liu L (2019). Therapeutic targeting of BRD4 in head neck squamous cell carcinoma. Theranostics.

[20] Zhang W, Ge H, Jiang Y, Huang R, Wu Y, Wang D, Guo S, Li S, Wang Y, Jiang H (2020). Combinational therapeutic targeting of BRD4 and CDK7 synergistically induces anticancer effects in head and neck squamous cell carcinoma. Cancer Lett.

[21] Webber LP, Yujra VQ, Vargas PA, Martins MD, Squarize CH, Castilho RM (2019). Interference with the bromodomain epigenome readers drives p21 expression and tumor senescence. Cancer Lett.

[22] Wang L, Wu X, Huang P, Lv Z, Qi Y, Wei X, Yang P, Zhang F (2016). JQ1, a small molecule inhibitor of BRD4, suppresses cell growth and invasion in oral squamous cell carcinoma. Oncol Rep.

[23] Slavish PJ, Chi L, Yun M-K, Tsurkan L, Martinez NE, Jonchere B, Chai SC, Connelly M, Waddell MB, Das S (2020). Bromodomain-selective BET inhibitors are potent antitumor agents against MYC-driven pediatric cancer. Cancer Res.

[24] Sun L, Fan G, Shan P, Qiu X, Dong S, Liao L, Yu C, Wang T, Gu X, Li Q (2016). Regulation of energy homeostasis by the ubiquitin-independent REGγ proteasome. Nat Commun.

[25] Antao AM, Tyagi A, Kim K-S, Ramakrishna S (2020). Advances in deubiquitinating enzyme inhibition and applications in cancer therapeutics. Cancers.

[26] Zhang W, Qiu W (2020). OTUB1 recruits tumor infiltrating lymphocytes and is a prognostic marker in digestive cancers. Front Mol Biosci.

[27] Niu Z, Li X, Feng S, Huang Q, Zhuang T, Yan C, Qian H, Ding Y, Zhu J, Xu W (2020). The deubiquitinating enzyme USP1 modulates ERα and modulates breast cancer progression. J Cancer.

[28] Dwane L, O'Connor AE, Das S, Moran B, Mulrane L, Pinto-Fernandez A, Ward E, Blümel AM, Cavanagh BL, Mooney B (2020). A functional genomic screen identifies the deubiquitinase USP11 as a novel transcriptional regulator of ERα in breast cancer. Can Res.

[29] Li W, Shen M, Jiang Y-Z, Zhang R, Zheng H, Wei Y, Shao Z-M, Kang Y (2020). Deubiquitinase USP20 promotes breast cancer metastasis by stabilizing SNAI2. Genes Dev.

[30] Wang L, Li M, Sha B, Hu X, Sun Y, Zhu M, Xu Y, Li P, Wang Y, Guo Y (2021). Inhibition of deubiquitination by PR-619 induces apoptosis and autophagy via ubi-protein aggregation-activated ER stress in oesophageal squamous cell carcinoma. Cell Prolif.

[31] Cui Z, Kang H, Grandis JR, Johnson DE (2020). CYLD alterations in the tumorigenesis and progression of human papillomavirus-associated head and neck cancers. Molecular cancer research : MCR.

[32] Orcutt SJ, Wu J, Eddins MJ, Leach CA, Strickler JE (2012). Bioluminescence assay platform for selective and sensitive detection of Ub/Ubl proteases. Biochim Biophys Acta.

[33] Zhang Q, Chen S, Yang M, Wang C, Ouyang Y, Chen X, Bai J, Hu Y, Song M, Zhang S (2020). Forkhead promotes EMT and chemoresistance by upregulating lncRNA CYTOR in oral squamous cell carcinoma. Cancer Lett.

[34] Yu W, Chen Y, Putluri N, Coarfa C, Robertson MJ, Putluri V, Stossi F, Dubrulle J, Mancini MA, Pang JC (2020). Acquisition of cisplatin resistance shifts head and neck squamous cell carcinoma metabolism toward neutralization of oxidative stress. Cancers.

[35] Garcia-Mayea Y, Mir C, Carballo L, Castellvi J, Temprana-Salvador J, Lorente J, Benavente S, García-Pedrero JM, Allonca E, Rodrigo JP (2020). TSPAN1: a novel protein involved in head and neck squamous cell carcinoma chemoresistance. Cancers.

[36] Zhu H, Yan F, Yuan T, Qian M, Zhou T, Dai X, Cao J, Ying M, Dong X, He Q (2020). USP10 promotes proliferation of hepatocellular carcinoma by deubiquitinating and stabilizing YAP/TAZ. Can Res.

[37] Xu P, Xiao H, Yang Q, Hu R, Jiang L, Bi R, Jiang X, Wang L, Mei J, Ding F (2020). The USP21/YY1/SNHG16 axis contributes to tumor proliferation, migration, and invasion of non-small-cell lung cancer. Exp Mol Med.

[38] Wu X, Liu D, Tao D, Xiang W, Xiao X, Wang M, Wang L, Luo G, Li Y, Zeng F (2016). BRD4 regulates EZH2 transcription through upregulation of C-MYC and represents a novel therapeutic target in bladder cancer. Mol Cancer Ther.

[39] Dong X, Hu X, Chen J, Hu D, Chen LF (2018). BRD4 regulates cellular senescence in gastric cancer cells via E2F/miR-106b/p21 axis. Cell Death Dis.

[40] Qin ZY, Wang T, Su S, Shen LT, Zhu GX, Liu Q, Zhang L, Liu KW, Zhang Y, Zhou ZH (2019). BRD4 promotes gastric cancer progression and metastasis through acetylation-dependent stabilization of snail. Cancer Res.

[41] Shi J, Wang Y, Zeng L, Wu Y, Deng J, Zhang Q, Lin Y, Li J, Kang T, Tao M (2014). Disrupting the interaction of BRD4 with diacetylated Twist suppresses tumorigenesis in basal-like breast cancer. Cancer Cell.

[42] Pastori C, Kapranov P, Penas C, Peschansky V, Volmar CH, Sarkaria JN, Bregy A, Komotar R, St Laurent G, Ayad NG (2015). The Bromodomain protein BRD4 controls HOTAIR, a long noncoding RNA essential for glioblastoma proliferation. Proc Natl Acad Sci U S A.

[43] Andrews FH, Singh AR, Joshi S, Smith CA, Morales GA, Garlich JR, Durden DL, Kutateladze TG (2017). Dual-activity PI3K-BRD4 inhibitor for the orthogonal inhibition of MYC to block tumor growth and metastasis. Proc Natl Acad Sci USA.

[44] Leonard B, Brand TM, O'Keefe RA, Lee ED, Zeng Y, Kemmer JD, Li H, Grandis JR, Bhola NE (2018). BET inhibition overcomes receptor tyrosine kinase-mediated cetuximab resistance in HNSCC. Cancer Res.

